# Equine grass sickness in italy: a case series study

**DOI:** 10.1186/s12917-021-02966-y

**Published:** 2021-08-06

**Authors:** Fulvio Laus, Jacopo Corsalini, Maria Teresa Mandara, Marilena Bazzano, Alice Bertoletti, Rodolfo Gialletti

**Affiliations:** 1 School of Bioscences and Veterinary Medicine, University of Camerino, Matelica, MC Italy; 2 Pratictioner, Perugia, Italy; 3 Department of Veterinary Medicine, University of Perugia, Perugia, Italy

**Keywords:** Grass sickness, Equine Dysautonomia, horse, intestinal dysmotility, pasture

## Abstract

**Background:**

Equine grass sickness (EGS) has been reported in several European and extra-European countries. Despite this, no scientific paper about clinical cases of EGS in Italy has been published. EGS is a disease affecting almost exclusively horses kept on pasture, characterized by clinical signs related to lesions in autonomic nervous system (ANS), particularly in the enteric nervous system (ENS). According to clinical presentation, acute, subacute and chornic syndromes can be observed, with various sympthoms including dullness, anorexia, dysphagia, drooling of saliva, tachycardia, ptosis, patchy sweating and muscle fasciculations. In horses affected by acute forms, mild to moderate abdominal pain and large volumes of nasogastric reflux can be observed. The etiology is still speculative and many hypothesis have been proposed to explain the pathogenesis.

**Case presentation:**

The present study describes four cases of EGS (one subacute and three chronic forms) occurred in Central Italy during early spring. In all the cases included in the study, the prognosis was poor and the horses were euthanized. The diagnosis was confirmed by histological examination of ANS or ENS. In two cases, *in vivo* diagnosis was obtained by histological examination of enteric bioptic samples collected during laparoscopy.

**Conclusions:**

EGS in Italy could be underdiagnosed and incidence understimated. Greater awareness should be applied in Italy for the inclusion of EGS in differential diagnosis for horses presenting clinical signs of abdominal pain associated or not with gastric reflux and muscular fasciculation. All the cases in this study concerned horses kept in the same pasture, confirming a possible premise-linked and management-linked factors on the ethiopathogenesis of EGS. The age of horses ranged from 2 to 6 years, that is consistent with the risk factor age for EGS (from 2 to 7 years of age). Previous suspected EGS diagnosis in the same livestock and recent cool dry weather were considered additional potential risk factors.

**Supplementary Information:**

The online version contains supplementary material available at 10.1186/s12917-021-02966-y.

## Background

Equine grass sickness (EGS) is a well known disease whose name derives from the close association between clinical signs and grazing activity. In fact, the disease seems to affect almost exclusively horses at pasture [1]. EGS is also known as Equine Dysautonomia because the majority of clinical signs are related to lesions in the enteric nervous system (ENS) and autonomic nervous system (ANS). However, clinical signs due to central nervous system pathological changes have been reported as well [2].

Three different syndromes can be recognized on the basis of the clinical signs, that are severe in the acute form, moderate in the subacute form, and mild in the chronic form [3].

Common clinical signs consist in dullness, anorexia, dysphagia, drooling of saliva and tachycardia [1, 3]. Ptosis, patchy sweating and muscle fasciculations can also be present in all forms of EGS [4, 5]. Acute EGS cases present mild to moderate abdominal pain and large volumes of gastric reflux [4, 5].

Clinical signs of subacute forms of EGS are similar to acute cases, but less severe [1]. The most significant features of chronic forms of EGS are weight loss, rhinitis sicca, the characteristic ‘tucked up’ abdominal silhouette [1] and a base-narrow stance [1, 4].

Rectal examination reveals distension of small intestine in acute EGS, and secondary large colon and caecal impactions in subacute EGS, by reflecting the intestinal dysmotility that results from neuronal loss in the ENS [6]. Persistent gastric reflux or the presence of firm colonic impactions are generally associated with a poor prognosis [6].

Clinical classification of EGS is rather arbitrary since clinical forms may overlap each other [3]. However, differentiating acute, subacute and chronic forms can be useful when expressing an actual prognosis, as some patients with chronic forms of EGS can survive whereas acute and subacute cases, usually lead to a fatal outcome [1, 3].

Histopathologic examination of intestinal submucosal and myenteric plexi and autonomic (vertebral and paravertebral) ganglia reveal typical changes of neurons including loss of Nissl substance, eccentric or pyknotic nuclei, cell swelling and cytoplasmic vacuolation, and intracytoplasmic spheroids bodies (axonal dystrophy) [3].

Etiology is still speculative and many hypotheses have been proposed to explain the pathogenesis. Historical investigations suggested the role of alsike clover (*Trifolium hybridum*) ingestion [7], *Clostridium perfringens* enterotoxicity [8–10], insect vectors [11], fungi [12] and *C. botulinum* toxicoinfection [7]. The role of ingested soil-borne agent able, under specific conditions, to produce or liberate a putative neurotoxin is supported by epidemiological studies [1]. The hypothesis of a climatic influence on etiologic agent exposure is supported by evidence like the seasonality of EGS, the association with grazing activity, and the geographic and temporal clustering of the disease [3].

EGS was firstly reported in Eastern Scotland [13] and then throughout the UK [1]. The disease has also been reported in several European countries, including Czech Republic [14], Belgium [15], Hungary [16, 17], Austria [18], Switzerland [19], Holland [20], Germany [21], France [22], Denmark [23] and Cyprus [24]. In the Falkland Islands [25] and Australia [26], suspected cases have also been reported. *Mal seco* is a disease of the South of America clinically and pathologically similar to EGS [27–30]. EGS has been recently described in a mule in the USA [31].

Albeit independently and occasionally diagnosed since the 1970 s, no scientific evìdence of EGS cases occurring in Italy has been published so far. The present report describes clinical signs and diagnosis of grass sickness in four horses reared for leisure activity in a farm located in Central Italy (43°40’20"64 N, 12°2’31"92 E; 433 m above sea level).

Clinical cases number 1, 2 and 3 were hospitalized at “masked for review” in March 2020, case number 4 in April 2020.

## Cases presentation

### Case 1

A 2-year-old Haflinger filly was admitted after one week from the onset of clinical signs including mild anorexia, depression, rapid weight loss and stop of stool production. The filly was kept on pasture with a group of similar breed horses. Two of them had suffered from slight colic signs few days before and had died in few hours.

At presentation, the filly was cachectic and had ‘tucked up appearance’ to the ventral abdomen. Depression of mental status was evident, and head and neck were carried down. Muscle weakness, muscular fasciculations, and base narrow stance were also observed. Clinical examination revealed bilateral palpebral ptosis, dry and congested ocular and oral mucous membranes, including the nasal mucosa (*rhinitis sicca*), noisy breathing, and slight dehydration. Hearth rate was elevated (60 bpm) and rectal temperature was normal (37.5 °C). Slow and prolonged mastication without drooling was present. Signs associated with cranial nerves deficits were not found. Colic pain was absent but abdominal sounds were reduced. Rectal examination and nasogastric intubation were unremarkable. Ultrasound examination revealed reduced motility of both small and large intestine. Abdominocentesis was performed and peritoneal fluid analysis was within the normal limits. Blood work was unremarkable.

A presumptive diagnosis of EGS was made and early therapeutic treatment consisting of fluid therapy and flunixin meglumine (0.5 mg/Kg IV twice a day) was administred. In addition, highly palatable diet was provided, and half an hour in-hand walking twice a day was performed.

On day 2, the work up to investigate weight lost syndrome included complete dental examination, fecal examination, gastroscopy, and oral glucose tolerance test (OGGT). Considering low glucose peak at 120 min, partial malabsorption was the only abnormality detected.

On day 3 and 4 clinical signs worsened, the filly became anorexic, depressed, and fecal production was inconsistent. Partial parental nutrition consisting in 5 % glucose, 8,5 % aminoacids and 1 ml/45 kg vitamins (thiamine 12.5 mg/ml; niacinamide 12.5 mg/ml; pyridoxine 5 mg/ml; d-panthenol 5 mg/ml; riboflavin 2 mg/ml; cyanocobalamin 5 mg/ml) was added to fluid therapy. Based on non-diagnostic histological results, exploratory laparotomy was proposed but refused by the owner due to financial constrains.

On day 7, further worsening of clinical conditions occurred, the filly was extremely weak and with no interest in feeding. Therefore, on day 8 the owners required euthanasia for ethical concerns and agreed to necropsy.

At the *post mortem* examination, signs of rhinitis without substantial exudate and diffuse catarrhal colityphlitis were the only relevant findings. Tissue samples from ileum, cranial cervical and cranial mesenteric ganglia were fixed in 10 % buffered formalin for routine histological examination.

**Fig. 1:**
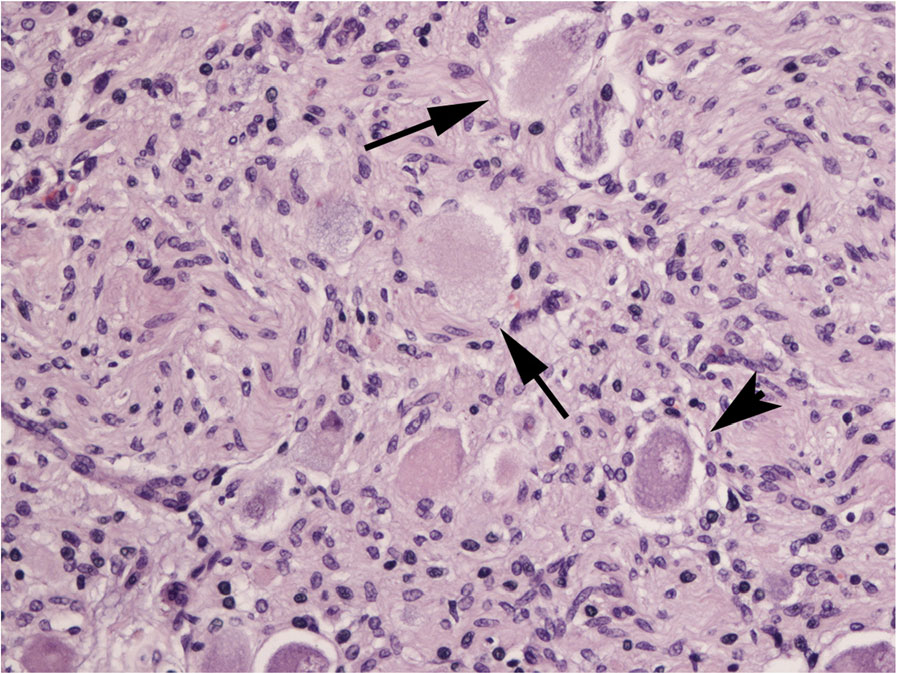
Case 1. Cranial mesenteric ganglion. A number of ganglion cells consist in *ghost neurons* (arrows), characterized by homogeneous eosinophilic cytoplasm and total loss of Nissl substance (complete chromatolysis). A marked reactivity of satellite cells is also present (glial scar). Normal ganglion cell (arrowhead) (FFPE, H&E, x20)

At histology, cranial cervical and cranial mesenteric ganglia showed severe loss of Nissl substance in numerous ganglion cells in association with increased number of satellite cells. Based on clinical evidence a diagnosis of chronic EGS was done (Fig. 1).

### Case 2

A 5-year-old Maremmana mare was referred with a history of loss of appetite, dullness and mild colic signs in the last two days. Actually, the first signs had been observed a week before worsening up to presentation. One of the horses kept on the same pasture had shown similar signs and had died abruptly.

At presentation, tachicardia (68 bpm) and decreased gut motility have been reported. Colic pain was absent but the horse was dull, anorexic and dehydrated. Rectal temperature was normal (37.8 °C), mucous membranes were dry and capillary refill time was delayed (3 s). Rectal examination revealed the presence of a live and active fetus of 3–5 months and a firm mass in the caudal part of the abdomen consistent with colonic impaction. Small amount of dark brown and mucoid feces were present in the rectum. At nasogatric intubation no reflux was found. Abdominal ultrasound findings included reduced motility of small and large intestine, non-distended fluid-filled stomach, and large non-sacculated ascending colon. Ectasic vessels were not present and anechoic peritoneal fluid was slightly increased and easily detectable next to the xiphoid apophysis of the sternum. Peritoneal fluid analysis was unremarkable.

Hematology was within normal limit. No significant clinical abnormality was detected on serum chemistry except for an increase in creatinine and urea values (Creatinine: 5.15 mg/dl, normal range 1.2–1.9 mg/dl; Urea: 197 mg/dl, normal range 10–24 mg/dl), total protein content (Serum Total protein: 9.2 g/dl, normal range 5.7–7.9 g/dl) and blood lactate concentration (Lactate: 3.3 mmol/l, normal range 1.1–1.7 mmol/L) consistent with prerenal azotemia due to dehydration.

A presumptive diagnosis of large colon impaction was made and medical therapy, consisting of fluid administration (40 ml/kg/day of ringer lactate) and mineral oil 2–4 L / 500-kg BW by nasogastric tube, was applied.

On day 2 and 3 the mare was still depressed, anorexic, tachicardic (60–70 bpm), with further reduction in intestinal motility. Muscles fasciculation and bilateral ptosis were reported.

**Fig. 2:**
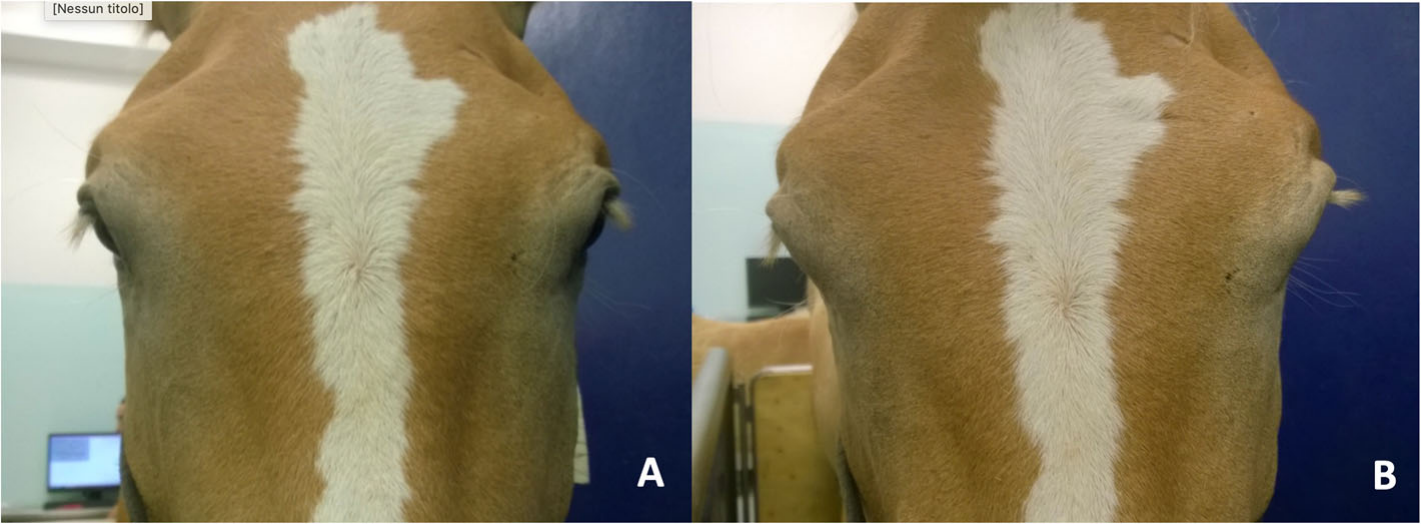
Case 3. before (**A**) and after (**B**) topical administration of 0.5 % phenylephrine on the left eye. Notice the reversal of ptosis in the treated eye

A phenylephrine test was performed by topical application of 0.5 % phenylephrine ophthalmic solution to the left conjunctival sac (Visumdriatic®, Visufarma SpA Rome). The right eye was used as a control. Temporary reversal of ptosis was observed on the treated eye after 30 min, confirming neurogenic Mūller superior tarsal muscle paralysis as the mechanism underlying the ptosis [32] (Fig. 2).

At transrectal palpation, although colon impaction was still present, greenish cow-like malodorous feces were detected in the rectal ampulla. Ultrasound examination revealed a fluid distended stomach and non-distended hypomotile small intestine. Fetus was live and active. On day 2, nasogastric tube passed through and 13 L of net reflux material were obtained in 12 h. No reflux was produced on day 3. Blood work revealed leukocytosis (12.300 wbc/ µL, reference range 5.4–14.3 wbc/µL) and fibrinogen content was slightly above the reference values (6.1 g/l, reference range 1–4 g/l). Urea and creatinine were returned within normal limits but total calcium (8.3 mg/dl, normal range 11.2–13.6 mg/dl) and potassium (2.1 mmol/l, normal range 2.4–4.7 mmol/l) were below reference values. KCl 30 mmol/l of polyionic solution and Ca gluconate 40 % 0,2 ml/kg were added to the ongoing fluid therapy (40 ml/kg/day of ringer lactate) for maintenance requirement.

On day 4 the mare showed moderate intermittent colic pain despite colonic impaction was no more detectable at rectal palpation. The mare interest for food improved but extreme dysphagia along with with patchy sweating and muscles fasciculation were observed during mastication. Serum chemistry and electrolyte (aspartate aminotrans- ferase (AST), gamma glutamyltransferase (GGT), creatine phosphokinase (CPK), total protein (TP), albumin (Alb), urea, creatinine (Crea), glucose (Glu), alkaline phosphstase (ALP), lactate dehydrogenase (LDH), total bilirubin (TB), direct bilirubin (DB), triglycerides (Tryg), cholesterol (Chol), calcium, phosphorus, magnesium, sodium, potassium, and chlorine) were within normal limits.

At night, between day 4 and day 5, the mare begun to show severe abdominal pain and congested mucous membranes. Blood lactate rised up (6.0 mmol/l). Transrectal examination was unremarkable and no reflux was obtained at nasogastric intubation. Ultrasonographic findings consisted of diffuse non-dilated hypomotile small intestine. The response to treatment (xilazine 0.4 mg/kg; flunixin meglumine 1.1 mg/kg) was poor and surgical treatment was refused by the owner due to financial constrains. The mare was humanly submitted to euthanasia.

Post mortem examination revealed a moderate right dorsal colon and descending colon impaction. Small intestine, particularly jejunum, was distended. Stomach content include *Gasterophilus spp.* larvae. Liver size was slightly increased. Fetus and fetus membranes were normal compared to the gestational age.

Multiple tissue specimens from all major organs were collected and fixed in 10 % buffered formalin, for routine histological staining. Histological findings consistent with EGS were observed in cranial and caudal mesenteric ganglia as well as in myenteric plexi (Additional file 1). As for gut, the lesions were more severe throughout the small intestine. Abnomal accumulation of lipofuscine in ganglion cells was also observed (Additional file 2).

### Case 3

A 5-year-old Haflinger mare was referred with a history of 12 h dullness, anorexia and abdominal pain unresponsive to medical therapy (flunixin meglumine, 1.1 mg/Kg, twice a day). At presentation, moderate signs of abdominal pain were present. The horse was dull, anorexic, dehydrated, tachycardic (68 bpm), and presented bilateral ptosis. Dry, sub-icteric mucous membranes and slightly elevated rectal temperature (38.1 °C) were also reported. Intestinal sounds were completely absent and the mare presented a base-narrow (elephant on a tub) stance.

Rectal examination revealed a hard ascending colonic impaction.

At abdominal ultrasound anechoic peritoneal fluid was slightly increased, small intestine and ascending colon were distended and hypomotile, stomach was full of ingesta and fluid but not distended. Nasogastric intubation was unsuccessful since the lower esophageal sphincter was completely closed preventing the passage of the tube.

Blood work revealed an increased packed cell volume (30 %) and total protein concentration (9 g/dl). Blood lactate was 2.8 mmol/l. Peritoneal fluid analysis was unremarkable.

Ptosis resulted to be positive to phenylephrine test.

Since the horse was kept on the same pasture of the horses for which equine grass sickness had been previously diagnosed, a laparotomy was performed in order to both obtain an ileal biopsy and solve the impaction.

The day after surgery, the horse was dull and showed slight signs of pain. Four liters of reflux were obtained by nasogastric intubation, and further 5 L were recovered five hours later.

On day 3 and 4 the horse was still dull and anorexic. Heart rate was increased (65 bpm) and abdominal sounds were absent with no stool production. On day 3 gastric reflux was 3 L, and on day 4 it was 4 L.

**Fig. 3:**
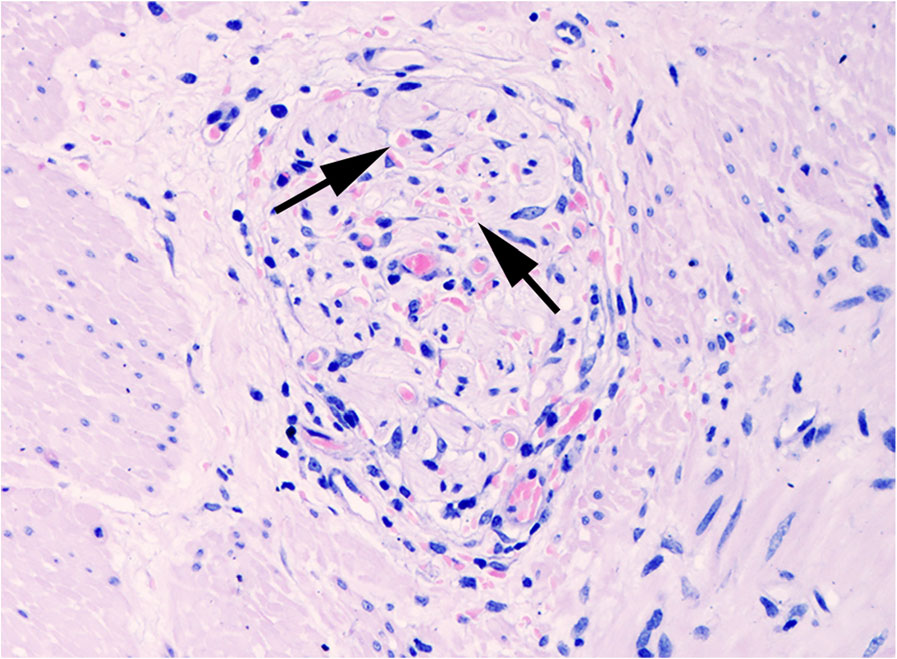
Case 3. Ileum. Myenteric plexus. Complete loss of ganglion cells associated with marked reaction by ganglion stromal cells. Small spheroid bodies (allows) are also scattered in the ganglion. (FFPE, H&E, x20)

Histological results of ileal biopsy (Fig. 3) were consistent with diagnosis of EGS.

Due to the clinical condition and worsening of colic pain, the horse was euthanized and submitted to necropsy in accordance with owner’s request. At necropsy, a large amount of ingesta was present inside the stomach and colon. Small amount of feaces covered by mucus were found in the small colon.

Post-mortem histological examination performed on ENS and cranial mesenteric ganglia confirmed findings consistent with EGS.

### Case 4

A 6-year-old Haflinger mare was referred along with a 45-day-old foal. The mare was dull and anorexic, showing mild colic pain, base-narrow stance, bilateral ptosis, increased heart rate (90 bpm). Intestinal sounds were absent. A few mucus covered black faeces were present in the rectum, and a hard constipation was detected in the pelvic flexure.

No significant change was detected at blood work (complete blood count and same serum chemistry parameters and electrolyte as in case 2) and peritoneal fluid analysis was unremarkable.

At abdominal ultrasound, distended small intestine was found. Phenylephrine eye test was positive.

Therapy for constipation (40 ml/kg/day of ringer lactate and mineral oil 2–4 L / 500-kg BW by nasogastric tube) was started but on day 2 the mare was still anorexic and the clinical condition did not improve. No significant changes were found at gastroscopy.

**Fig. 4:**
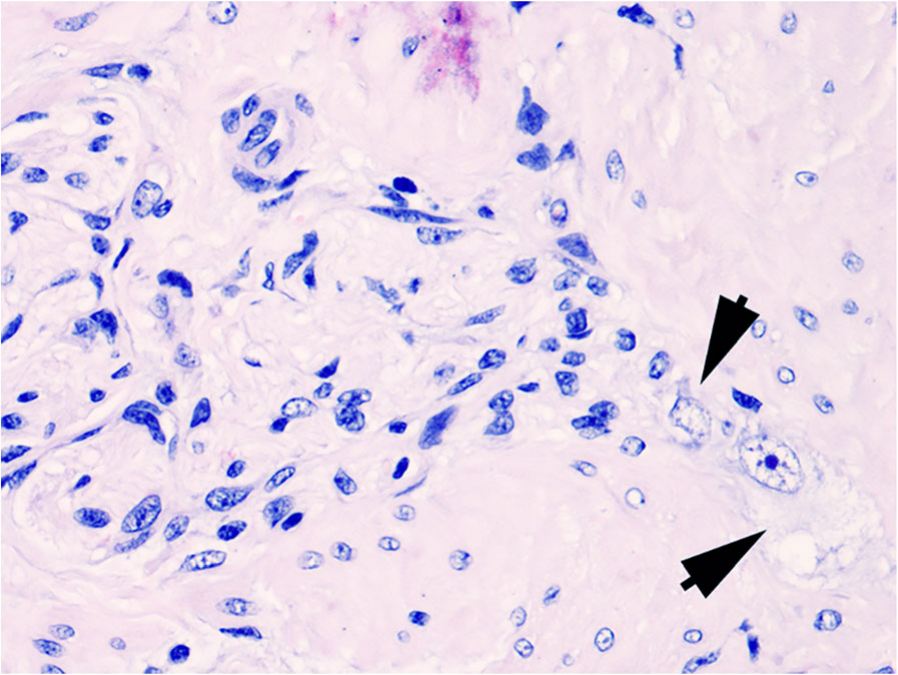
Case 4. Ileum. Myenteric plexus. Severe depletion of ganglion neurons. Two perikaryons are still recognizable (arrow heads) compared to increased number of ganglion stromal cells. (FFPE, H&E, x40)

Based on the history and clinical presentation, EGS was suspected and a standing laparoscopy was planned to collect full thickness ileal biopsy, as previously described [33]. Despite a slight improvement of the appetite and of the abdominal pain condition, on day 6, two days after surgery, the mare became again anorexic and dull, the abdominal sounds were still absent and no stool production was observed. Since histological findings of ileal biopsy were consistent with EGS (Fig. 4), the mare was euthanized. Histology of ENS and mesenteric ganglia sampled at post-mortem examination confirmed the diagnosis of EGS (Additional file 3).

## Discussion and conclusions

Clinical features of the presented cases were consistent with diagnosis of chronic (case 1) or subacute EGS (case 2, 3 and 4). Bilateral ptosis was confirmed to be almost invariably present in EGS cases, especially in subacute form [3]. All the cases here described concerned horses kept on pasture, as commonly reported in the literature for cases of EGS [1]. Furthermore, all horses lived on the same pasture confirming a possible premise-linked and management-linked factors on the ethiopathogenesis of EGS [3]. The age of horses ranged between 2 and 6 years, that falls within the risk age for EGS, reported to be from 2 to 7 years [1]. The occurrence of previous suspected EGS cases in the same geographical area, and recent climatic changes with cool dry weather in the early spring (cases 1 and 4), might be considered additional potential risk factors, as reported in the literature [34]. These evidences further support the potential involvement of pasture-derived mycotoxins in the etiopathogenesis of EGS closely related to the geographic and temporal clustering of cases, and to the seasonality of the disease [3, 35].

In cases 3 and 4 a standing laparoscopy for ileal tissue sampling was performed to achieve a definitive diagnosis. Indeed, laparoscopic guided biopsies should be a useful tool to confirm suspected cases of EGS, and to assess the grade of lesions for prognosis perspectives [1].

Chronic cases of EGS can sometimes recover, but when anorexia lasts for several consecutive days, as occurred in case 1, the prognosis is poor [3]. Subacute forms of EGS (cases 2, 3 and 4) were confirmed to be fatal as described in literature [3].

To date, in Italy cases of EGS are trackable in national meetings of 1970 s and/or included in personal not published data. Based on personal experience we are convinced that in our Country EGS is a pathological condition more deeply-rooted than we aware worthy of being much more traced and investigated.

To the author’s knowledge this is the first report of equine grass sickness in Italy. According to our findings, this disease should be included among the differential diagnoses in all horses kept on pasture and presenting clinical evidence of intestinal dismotility in Italy, as well. Environmental and climatic conditions typical of Central Italy should be strongly considered as risk factors also in other geographical regions of the Country.

## Supplementary information

Additional file 1: **Figure 1 Supplementary.** Case 2. Cranial cervical ganglion. Severe neuronal chromatolysis and satellitosis, along with occasional lymphocytic cells (arrow head). (FFPE, Cresil violet, x20).

Additional file 2: **Figure 2 Supplementary.** Case 2. Caudal mesenteric ganglion. A number of ganglion cells contain abnormal accumulation of yellowish pigments consistent with lipofuscins (FFPE, H&E, x40).

Additional file 3: **Figure 3 Supplementary.** Case 4. Cranial mesenteric ganglion. Many ganglion cells show complete loss of Nissl’s substance replaced by a diffuse eosinophilic cytoplasm. In these cells the nucleus is not apparent. A marked gliosis is associated with neuronal degeneration (FFPE, H&E, x10)

## Data Availability

All data generated or analyzed during this study are included in this published article.

## Abbreviations

EGS: Equine Grass Sickness
ANS: Autonomic Nervous System
ENS: Enteric Nervous System
OGGT: Oral Glucose Tolerance Test
