# Correction to: Equine grass sickness in Italy: a case series study

**DOI:** 10.1186/s12917-022-03195-7

**Published:** 2022-03-22

**Authors:** Fulvio Laus, Jacopo Corsalini, Maria Teresa Mandara, Marilena Bazzano, Alice Bertoletti, Rodolfo Gialletti

**Affiliations:** 1 School of Bioscences and Veterinary Medicine, University of Camerino, Matelica, MC Italy; 2 Pratictioner, Perugia, Italy; 3 Department of Veterinary Medicine, University of Perugia, Perugia, Italy

**Correction to: BMC Vet Res 17, 264 (2021)**

**https://doi.org/10.1186/s12917-021-02966-y**

Following the publication of the original article [1], it was noticed that authors’ first names and family names were interchanged. Corrected names are shown in the author section above.

The original article has been corrected.
