# Supplementary material for: Equine grass sickness in italy: a case series study
Source: BMC Vet Res. 2021 Aug 6;17:264. doi: 10.1186/s12917-021-02966-y (PMC8343987; doi:10.1186/s12917-021-02966-y)
Supplement: Supplementary file 1 — Figure 1 Supplementary. Case 2. Cranial cervical ganglion. Severe neuronal chromatolysis and satellitosis, along with occasional lymphocytic cells (arrow head). (FFPE, Cresil violet, x20). [file 12917_2021_2966_MOESM1_ESM.docx]

Additional file 1: **Figure 1 Supplementary**. Case 2. Cranial cervical ganglion. Severe neuronal chromatolysis and satellitosis, along with occasional lymphocytic cells (arrow head). (FFPE, Cresil violet, x20)
