# Supplementary material for: Equine grass sickness in italy: a case series study
Source: BMC Vet Res. 2021 Aug 6;17:264. doi: 10.1186/s12917-021-02966-y (PMC8343987; doi:10.1186/s12917-021-02966-y)
Supplement: Supplementary file 2 — Figure 2 Supplementary. Case 2. Caudal mesenteric ganglion. A number of ganglion cells contain abnormal accumulation of yellowish pigments consistent with lipofuscins (FFPE, H&E, x40). [file 12917_2021_2966_MOESM2_ESM.docx]

Additional file 2: **Figure 2 supplementary.** Case 2. Caudal mesenteric ganglion. A number of ganglion cells contain abnormal accumulation of yellowish pigments consistent with lipofuscins (FFPE, H&E, x40).
