# Supplementary material for: Equine grass sickness in italy: a case series study
Source: BMC Vet Res. 2021 Aug 6;17:264. doi: 10.1186/s12917-021-02966-y (PMC8343987; doi:10.1186/s12917-021-02966-y)
Supplement: Supplementary file 3 — Figure 3 Supplementary. Case 4. Cranial mesenteric ganglion. Many ganglion cells show complete loss of Nissl’s substance replaced by a diffuse eosinophilic cytoplasm. In these cells the nucleus is not apparent. A marked gliosis is associated with neuronal degeneration (FFPE, H&E, x10) [file 12917_2021_2966_MOESM3_ESM.docx]

Additional file 3: **Fugure 3 supplementary.** Case 4. Cranial mesenteric ganglion. Many ganglion cells show complete loss of Nissl’s substance replaced by a diffuse eosinophilic cytoplasm. In these cells the nucleus is not apparent. A marked gliosis is associated with neuronal degeneration (FFPE, H&E, x10).
